# The impact of poor metabolic health on aggressive breast cancer: adipose tissue and tumor metabolism

**DOI:** 10.3389/fendo.2023.1217875

**Published:** 2023-09-20

**Authors:** Barbara Mensah Sankofi, Estefania Valencia-Rincón, Malika Sekhri, Adriana L. Ponton-Almodovar, Jamie J. Bernard, Elizabeth A. Wellberg

**Affiliations:** ^1^ Department of Pathology, University of Oklahoma Health Sciences Center, Oklahoma City, OK, United States; ^2^ Department of Pharmacology and Toxicology, Michigan State University, East Lansing, MI, United States; ^3^ Nicolas V. Perricone Division of Dermatology, Michigan State University, East Lansing, MI, United States; ^4^ Department of Medicine, Michigan State University, East Lansing, MI, United States

**Keywords:** breast cancer, adipose, fibroblast growth factor, obesity, diabetes

## Abstract

Obesity and type 2 diabetes are chronic metabolic diseases that impact tens to hundreds of millions of adults, especially in developed countries. Each condition is associated with an elevated risk of breast cancer and with a poor prognosis after treatment. The mechanisms connecting poor metabolic health to breast cancer are numerous and include hyperinsulinemia, inflammation, excess nutrient availability, and adipose tissue dysfunction. Here, we focus on adipose tissue, highlighting important roles for both adipocytes and fibroblasts in breast cancer progression. One potentially important mediator of adipose tissue effects on breast cancer is the fibroblast growth factor receptor (FGFR) signaling network. Among the many roles of FGFR signaling, we postulate that key mechanisms driving aggressive breast cancer include epithelial-to-mesenchymal transition and cellular metabolic reprogramming. We also pose existing questions that may help better understand breast cancer biology in people with obesity, type 2 diabetes, and poor metabolic health.

## 1 Introduction

Breast cancer is the second most commonly diagnosed malignancy in women (1), with almost 300,000 new cases and 43,000 deaths expected in 2023 (2). Heritable mutations account for some breast cancer diagnoses; a well-known example of which are the BRCA1/2 genes. However, most breast cancers arise from environmental or lifestyle-associated exposures. Two major contributors to breast cancer risk and mortality, although not necessarily separable, are obesity and type 2 diabetes (T2D). Approximately 1.9 billion adults worldwide have a BMI over 25 kg/m^2^, and 650 million (13%) of these have obesity (3). In the US, 42.4% of adults have obesity (43% of men and 41.9% of women). Globally, approximately 10.5% of adults have diabetes (4) and up to 90% of these cases are T2D (5). Excess body weight is a prominent risk factor for T2D; approximately 90% of people with T2D have overweight or obesity (6, 7). Importantly, according to the Centers for Disease Control and Prevention, more than 1 in 3 Americans have prediabetes and more than 80% of these people do not know they have it. Some strategies that promote weight loss or improve metabolic function, such as bariatric surgery, calorie restriction, or metformin, are known to associate with reduced breast cancer risk, suggesting that impairments in whole body metabolism are reversible (8–10). Once tumors form, poor metabolic health is likely to influence breast cancer risk and progression through multiple mechanisms.

At diagnosis, breast tumors are histologically defined based on the presence of specific receptors. These categories include hormone receptor-positive (HR+), which express the estrogen or progesterone receptors (ER/PR) and are collectively referred to here as estrogen receptor-positive (ER+), human epidermal growth factor receptor positive (HER2+), or triple-negative breast cancer (TNBC), which lacks all of these receptors. Each subtype has distinct treatment and mortality profiles that depend in part on the stage of tumor at diagnosis (localized versus distant) and on the expression of HR or HER2. The disease-free and disease-specific survival rates for ER+, HER2+, and TNBC (2) illustrate the unique biology of these tumors. Aside from the histological subtypes, there are also molecular subtypes that are reflected in tumor gene expression profiles including luminal A, luminal B, HER2-enriched, basal-like, and normal breast-like (11, 12) that each have unique survival patterns (13). TNBC or basal breast cancer cases occur frequently in women under 40 and people of African ancestry. These cases are characterized by greater early relapse and advanced disease stage at diagnosis. While the TNBC subtype is often more aggressive than ER+ breast cancer, approximately 40% of ER+ tumors may be resistant to endocrine therapy. Out of these, 15-20% demonstrate intrinsic and early resistance to treatments (14–16). Intrinsic or *de novo* resistance to endocrine therapies can occur with low ER expression (17), while acquired resistance occurs through several mechanisms such as mutations in the ESR1 gene, loss of ER expression, alteration of transcriptional co-regulatory proteins, growth factor receptor activation, and metabolic reprogramming (18, 19). Nearly 50% of patients with TNBC develop resistance to chemotherapy, attributable in part to poor cellular differentiation and the presence of cancer stem cells that frequently self-renew to drive chemoresistance (20). Recurrent ER+ tumors are insensitive to endocrine therapy, similar to TNBC, suggesting that endocrine therapy-resistant ER+ breast cancer may be functionally similar to TNBC, with potentially overlapping risk factors and drivers.

In this review, we describe some common features of aggressive breast cancers such as TNBC and endocrine-resistant ER+ tumors. We discuss factors that characterize poor metabolic health and the influence these have on breast tumor biology, emphasizing adipose tissue. While there are many reported mechanisms through which obesity and T2D impact cancer, our work has identified the fibroblast growth factor signaling network (FGF/FGFR) as one common pathway connecting metabolic dysfunction to both TNBC and endocrine-resistant ER+ breast tumors and we describe those studies (21–26). We also examine the various ways tumor FGFR signaling may be altered and potential downstream effects, including epithelial to mesenchymal transition and altered tumor metabolism, that contribute to the hallmarks of aggressive breast cancer.

## 2 The impact of metabolic health on breast cancer risk and prognosis

### 2.1 Obesity

Postmenopausal breast cancer is one of 13 obesity-associated cancers (27). However, obesity is associated with a lower risk for premenopausal breast cancer (28). This seems counterintuitive, and many scientists are still searching for an explanation for this phenomenon (29). In addition to menopausal status, the impact of obesity on breast cancer risk depends on the tumor subtype. Prior to menopause, a high BMI is associated with a lower risk for ER+ breast cancer (30–33), which may explain the overall lower incidence of breast cancer associated with obesity in young women. As mentioned above, ER+ tumors make up the majority of diagnosed cases and likely drive the statistics that link obesity to breast cancer as a whole. The risk for TNBC prior to menopause is elevated with obesity (33, 34). Obesity is defined as a BMI30kg/m^2^ by the World Health Organization; however, one large study indicated that the elevated risk for breast cancer diagnosis is apparent in women with a BMI>25kg/m^2^, which includes those considered “overweight” (35). The risk for contralateral breast cancer diagnosis in women who received conserving surgical treatment is significantly elevated in women with overweight or obesity (36).

Some studies have shown a link between obesity and a higher risk of TNBC (34, 37) and premenopausal ER-negative breast cancer (32, 38). In the Carolina Breast Cancer Study, obesity was associated with increased incidence of TNBC in both premenopausal and postmenopausal women of African descent (39). Similarly, the Women’s Circle Health Study showed that high waist-to-hip ratio was associated with an increased risk of premenopausal breast cancer in African Americans after adjusting for BMI (40). Another study also reported that obese premenopausal women had an 82% increased risk of TNBC compared with women with high BMI (34). Also, a meta-analysis by Pierobon and colleagues showed that premenopausal obese women have a 42% higher risk of developing TNBC compared with women with normal BMI (41)

Obesity is associated with a shorter overall survival in women with breast cancer and many but not all studies report a significant negative effect of obesity on recurrence-free or breast cancer-specific survival (29, 42–47). The relationship between obesity and breast cancer specific survival may depend on a variety of factors, including tumor subtype, lymph node involvement, tumor stage, and years since menopause Importantly, obesity is associated with other morbidities such as CVD, which may influence survival more strongly than breast cancer progression. Consequently, the epidemiological data describing the effects of obesity, metabolic function, and their associated sequelae on breast cancer mortality are not yet as consistent as those for breast cancer risk. This may be due, in part, to underpowered studies that cannot assess each variable and its impact on breast cancer-specific survival, or due to the complex physiology associated with metabolic health in humans. Early studies with extensive follow-up may not have taken into consideration the complexity of breast cancer subtypes or the driving mechanisms that underlie the BMI-breast cancer link.

### 2.2 Type 2 diabetes

Multiple studies have investigated the effect of diabetes on breast cancer risk and progression (48, 49). According to one meta-analysis, women with diabetes have a 23% higher risk of developing breast cancer than those without diabetes (50). Furthermore, a greater percentage of women with diabetes have a more advanced stage of breast cancer than their non-diabetic counterparts (48). A different study reported that women with diabetes had a 27% greater risk for breast cancer, but this was decreased to 16% when studies were considered that controlled for BMI, indicating the importance of obesity in the link between T2D and breast cancer (51). Fasting insulin and glucose, which precede diabetes diagnosis, have been associated with a greater risk for breast cancer in women with a BMI over 26 kg/m^2^ (52). A similar impact of diabetes is seen on all-cause or overall mortality and on breast cancer-specific mortality. In most cases, the relationship between diabetes and all-cause mortality is stronger than that for cancer-specific mortality since diabetes is associated with other chronic conditions that can shorten lifespan. One meta-analysis found that diabetes is linked with a 37% and 17% increased risk of all-cause mortality and breast cancer-specific mortality, respectively (53). Another study revealed that compared to those without diabetes, breast cancer patients with pre-existing diabetes had a 51% shorter overall survival time and a 28% shorter disease-free survival (54). The Cancer Prevention Cohort Study II which recruited one million US adults reported a 16% increase in breast cancer-specific mortality and a 2-fold increased risk of all-cause mortality (55). Another retrospective study reported a two-fold greater breast cancer-specific mortality in women with T2D (56). Diabetes also significantly affects the treatment of choice of breast cancer patients. For example, younger women with both diabetes and breast cancer were more likely to undergo surgical resection than their non-diabetic counterparts (57). Women with insulin-treated diabetes were less likely to undergo axillary lymph node dissection relative to their non-diabetic counterparts. Older patients (<65 years) were less likely to receive radiotherapy than their non-diabetic patients of the same age bracket (58). T2D is diagnosed based on hemoglobin A1c levels, which become elevated after insulin resistance is established. The environment of hyperinsulinemia, often referred to as prediabetes, may occur long before pancreatic beta-cell failure that characterizes T2D; however, it is difficult to capture the link between this environment and breast cancer risk without screening criteria to define the prediabetic state.

### 2.3 Other informative individual variables

Beyond BMI, other host variables may better predict breast cancer risk than the amount of body fat and may indicate the importance of developing tools to routinely assess metabolic health before diabetes develops (25, 59). Adipose distribution often changes after menopause, when women experience more visceral adiposity or central obesity, reflected by a greater waist-to-hip ratio. Central obesity is associated with elevated risk of both ER+ and TNBC subtypes (32, 46). Adult weight gain (i.e. adipose expansion) is associated with elevated breast cancer risk in multiple studies (60–63). Metabolic health, defined by a variety of criteria, may be an independent predictor of breast cancer risk in some people. In several studies, postmenopausal women that were considered “metabolically unhealthy” using different approaches (e.g. high fasting insulin, high HOMA-IR, hepatic steatosis), had elevated breast cancer risk irrespective of BMI (64–66). However, a very recent study indicated no difference in postmenopausal breast cancer risk in women classified as metabolically healthy overweight/obese or as metabolically unhealthy lean using C-peptide measures (67). Deteriorating metabolic function may indicate the presence of prediabetes, which is characterized by sustained hyperinsulinemia. Insulin, a potent mitogenic and anabolic hormone, may support breast cancer progression through the activation of MAPK and PI3K pathways that lead to many pro-tumorigenic cellular changes. Elevated insulin is a feature of the “metabolically unhealthy” lean or obese phenotype, is often associated with visceral adiposity, and is linked to adult weight gain. According to the Women’s Health Initiative Study, higher levels of Insulin resistance in post-menopausal women are associated with higher breast cancer incidence and higher all-cause mortality after breast cancer. The role of insulin in regulating cancer growth has been recently reviewed (68). Currently, it remains to be established whether experimentally preventing hyperinsulinemia impacts breast cancer risk or progression.

## 3 The role of adipose tissue in aggressive breast cancer biology

The crosstalk between obesity, T2D, and breast cancer is intricate and mediated by multiple mechanisms including, inflammation, adipose tissue dysfunction, metabolic alterations, insulin, and hypoxia. Many of these mediators have been thoroughly reviewed elsewhere (68, 69). There are 13 obesity-associated cancers for which elevated risk is associated with a high BMI. Almost all of them, including liver, pancreas, endometrial, and certainly breast cancer, occur adjacent to or within adipose depots. The breast is composed of 90% adipose tissue, which contains many cell types including adipocytes, fibroblasts, vascular cells, and immune cells. Obesity and T2D have been implicated in adipose tissue dysfunction, which alters the cellular interactions within the breast that often support cancer therapy resistance. Generally, patients with obesity and/or T2D have poor responses to breast cancer therapies and greater mortality, as described above. As an endocrine organ, adipose tissue produces pro-inflammatory cytokines, adipokines, growth factors, and also aromatizes androgens into estrogens. These factors can alter the tumor microenvironment and promote aggressive breast cancer. Adipose tissue dysfunction leads to excessive production of adipokines such as leptin and adiponectin by the adipocytes (70). Low serum adiponectin levels were associated with a higher risk for metastasis, angiogenesis, and endocrine therapy resistance (71, 72). Besides adiponectin, obese individuals have a higher expression of leptin and its receptor which has been implicated in increasing the metastatic potential in the tumor microenvironment. Also, leptin has been shown to activate ERα signaling and increase aromatase activity leading to excessive proliferation and migration of tumor cells (73, 74). Knockdown of leptin in adipose-derived stromal cells co-cultured with ER+ breast cancer cells led to a reduction in tumor growth and expression of metastasis-related genes (75). Overall, leptin and adiponectin have opposing effects on breast tumorigenesis and their ratio may be modulated particularly in people with high BMI as it significantly increases breast cancer risk and metastasis.

### 3.1 Estrogen

After menopause, adipose tissue is a major source of estrogen in women, and women with obesity have elevated circulating and local levels of estrogens. The elevated levels of estrogen (estrone and estradiol) in women with obesity undoubtedly contribute to the greater breast cancer risk associated with a high BMI. Indeed, a recent study showed that, in women with BRCA mutations and a high BMI, estrogen biosynthesis was elevated compared to women with a low BMI, indicating a role for estrogen in DNA damage that contributes to breast cancer risk (76). However, aromatase inhibitors such as letrozole and anastrazole effectively reduce circulating estrogen levels by up to 98% in postmenopausal women, irrespective of BMI (77). Clinical studies testing higher aromatase inhibitor doses have found no added benefit to lowering estrogens in women with obesity (78) or to breast cancer outcomes in general (79, 80), suggesting that the mechanisms of obesity-associated breast cancer progression and mortality are estrogen-independent.

### 3.2 Inflammation

Both obesity and T2D influence pro-inflammatory immune cells implicated in breast cancer development and progression. Obesity is characterized by hypertrophic adipocytes that recruit macrophages and sustain chronic low-grade inflammation. The breast environment in women with obesity has been documented to have crown-like structures, which are named by the histological appearance of macrophages around dying adipocytes. These inflammatory foci are also present in women without obesity, but who are classified as metabolically unhealthy (81, 82), illustrating the potential limitation of using BMI to evaluate breast cancer risk on an individual level. Obesity promotes features of immune cell exhaustion in mice and humans (83, 84), but obesity is also associated with a better response to immune therapy, for example in retrospective studies of melanoma (85). Similar links are seen with T2D, where immune cells may be senescent and low-grade inflammation is present (86). In a small study of people with a genetic predisposition to diabetes (i.e. first-degree relatives of people with T2D) matched with controls for BMI, gender, and age, subcutaneous adipocyte hypertrophy was evident even prior to the development of obesity or overt T2D (87). Greater adipocyte size was associated with elevated fasting insulin, higher HOMA-IR, and proinflammatory cytokines such as IL1β and IL6 (87). Studies like these studies highlight the potentially tumor-promotional changes that could take place in the breast adipose microenvironment even before obesity or T2D develops. The intricate relationship surrounding obesity, metabolic dysfunction, and inflammation as it relates to breast cancer has been comprehensively reviewed by others (68, 81, 88).

### 3.3 Growth factors: emphasis on FGF/FGFR signaling

Within the breast tumor environment, growth factors such as EGF, VEGF, IGFs, and FGFs are produced by fibroblasts, adipocytes, and by cancer cells themselves. Each of these stimulates aggressive features of breast tumors and can drive resistance to breast cancer therapies. Deregulated growth factor signaling influences several cancer hallmarks by supporting cell proliferation, rendering cancer cells resistant to apoptosis, promoting vascularization, and stimulating invasion and metastasis.

Fibroblast growth factor receptor (FGFR) signaling is crucial for breast development, tissue homeostasis, malignant transformation, and metastasis. There are 22 FGF ligands (18 of which signal through receptors) and 4 FGFR genes (89). Alternative splicing generates multiple isoforms of FGFRs 1-3, while FGFR4 has only one isoform (89). The ligands have paracrine or endocrine functions, with multiple ligand/receptor interactions possible. Dysregulated FGF/FGFR production or activation has been implicated in breast cancer progression and aggressive breast cancer phenotypes. FGF signaling regulates normal mammary stem cells and gland development, illustrated by the reduction in mammary outgrowth in mice lacking FGFR1 and FGFR2 (90). Conversely, activation of FGFR1 in mammary epithelium causes alveolar proliferation and early features of transformation (91). FGF and FGFR DNA alterations have been described in a variety of cancers, including breast cancer. For example, FGFR1 is frequently amplified and overexpressed in luminal breast tumors (up to 27% of cases), while FGFR2 is amplified in TNBC, although less frequently. Overexpression of FGFs and FGFRs in breast cancer cells can lead to aberrant pathway activation, which is why clinical trials have focused on targeting FGFRs in patients (92). The downstream effects of FGF signaling include activation of PI3K/AKT, MAPK, PLC, and JAK/STAT networks. Each of these pathways elicits a variety of responses that encompass the hallmarks of cancer such as proliferation, survival, migration, invasion, and changes in gene expression profiles and cell differentiation, including EMT. FGF signaling can promote growth, metastasis, and stemness in a variety of breast cancer cell subtypes and preclinical models (reviewed in (92).

#### 3.3.1 FGFs from cells in the breast environment

FGFs are produced by breast cancer cells and by cells in the breast TME, including adipocytes and cancer-associated fibroblasts (CAFs). Obesity can impact each of the cells in the TME (25, 93). For example, one study found that adipose-derived fibroblasts from donors with obesity expressed elevated CAF markers compared to those from lean individuals. Co-culture of ER+ MCF7 cells with these obesity-associated fibroblasts facilitated proliferation and invasion more than those cultured with lean fibroblasts (94). Exposure of human fibroblasts to sera from women with obesity promoted pro-inflammatory changes that may impact signaling in breast cancer cells (95). In a mouse model of obesity, FGF1 expression was elevated in mammary adipose tissue after estrogen withdrawal-induced weight gain (23). Hypertrophic adipocytes secrete FGF1 as a proliferative signal to undifferentiated preadipocytes during weight gain (96). This normal signaling mechanism ensures healthy adipose tissue expansion through hyperplasia and new adipocyte formation, but can also support the proliferation of nearby breast cancer cells. ER+ tumors that continued to grow after estrogen loss had elevated levels of phosphorylated FGFR1, and inhibition of FGFR restored sensitivity of these tumors to estrogen deprivation (23). In subcutaneous adipose tissue biopsies taken from people with obesity before and after intentional weight gain, FGF1 expression increased significantly but only in those people who were classified as metabolically unhealthy. In human breast adipose, FGF1 expression positively correlated with BMI and with adipocyte size. In humans, high tumor levels of phosphorylated FGFR1 associated with a shorted disease-free survival after tamoxifen treatment and with an elevated BMI. An additional study found that circulating FGF2 was directly correlated with BMI in patients with breast cancer, and FGF2 treatment of obese tumor-bearing mice promoted resistance to anti-VEGF therapy (97). In TNBC (MDA-231), CAFs can stimulate FGFR1 signaling (98), and FGF2 from CAFs promotes cell migration and tumor growth through cancer cell FGFR1 (99). FGF2 produced by visceral adipose tissue stimulated growth of non-cancerous human skin cells and MCF10A human breast epithelial cells in soft agar and in low-attachment sphere cultures, which is a hallmark of malignant transformation (21, 22, 24). A pilot study using human serum in soft-agar MCF10A cultures showed that FGF2 signaling through FGFR1 may be necessary but not sufficient to promote sphere formation (100). There is compelling evidence demonstrating the role of FGF1 and FGF2 as potent angiogenic factors in mediating increased breast cancer risk and progression (92, 101, 102). One study showed that in ovariectomized or tamoxifen-treated mice, MCF7 cells overexpressing FGF1 displayed increased vascularization and enhanced metastatic potential (103). FGF2 was linked to obesity and elevated resistance to anti-VEGF therapy in a preclinical study. Its inhibition reduced vascular density and restored tumor susceptibility to anti-VEGF therapy in obese mice (97). In addition, crosstalk between FGF2 and VEGF synergistically amplifies breast tumor angiogenesis and metastasis (97, 104). These studies show that adipose-derived factors associated with obesity may not need to originate in the local tumor environment but can come from dysfunctional adipose around the body (25). Future research will fully define the FGF-mediated mechanisms that contribute to malignant transformation of ER-negative breast cells.

#### 3.3.2 FGF signaling in luminal breast cancers

**Figure 1:**
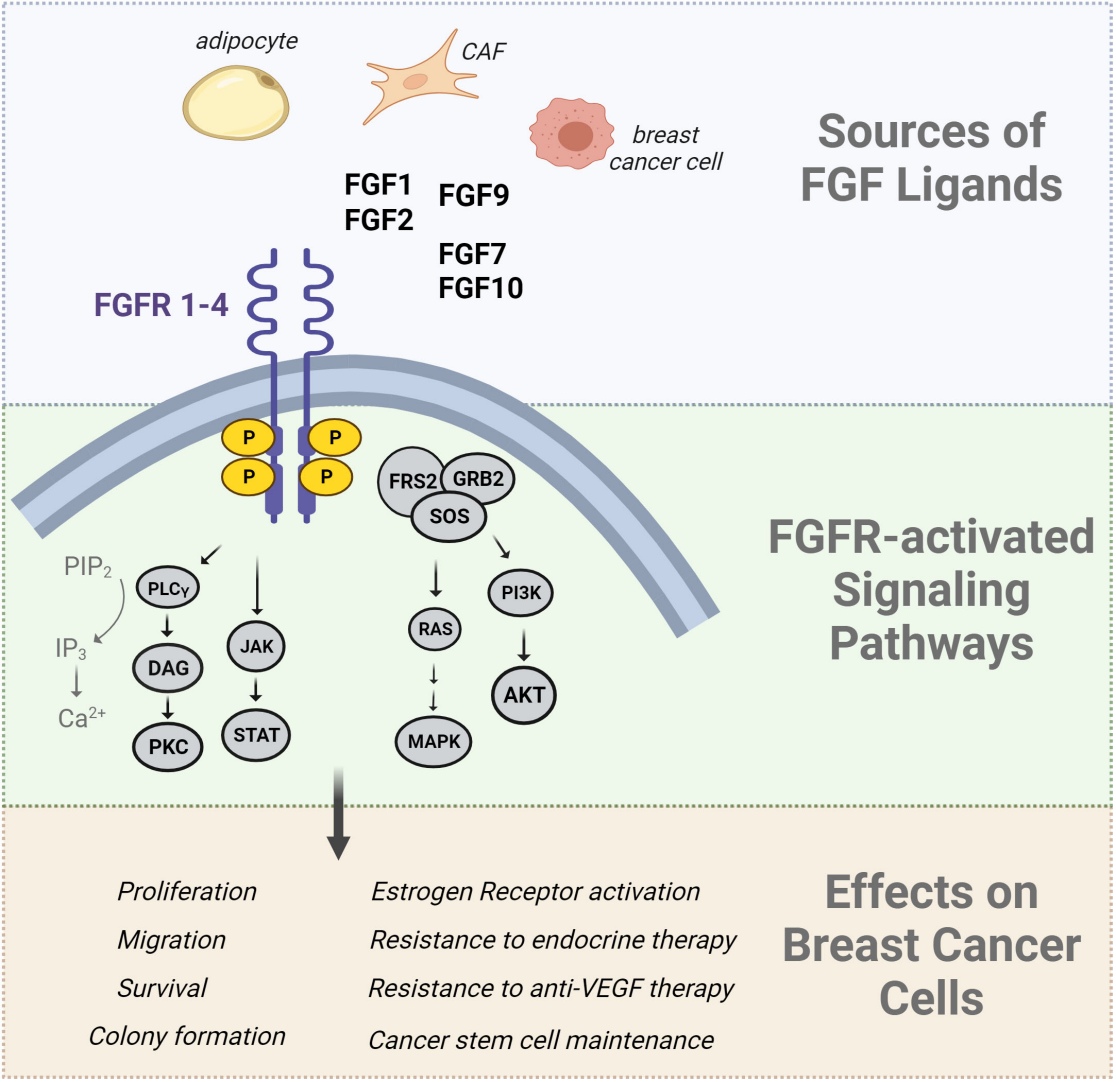
Summary of FGF effects in breast cancer cells. The Fibroblast Growth Factor (FGF) ligands are produced by a variety of cells including adipocytes, cancer associated fibroblasts (CAFs), and breast cancer cells. FGFs bind to FGF receptors, inducing receptor dimerization and transphosphorylation of its intracellular kinase domain. The FGFR signaling network activates downstream signal transduction pathways such as the RAS/MAPK pathway, PI3K/AKT pathway, PLCγ pathway, and JAK/STAT pathways. The downstream effects of these pathways include an increase in cell proliferation, migration, angiogenesis, and survival. Downstream-activated effector molecules of FGFR signaling can also activate the estrogen receptor in a ligand-independent manner which is implicated in endocrine therapy resistance.

Like other growth factor receptors, FGFR can stimulate ER activation independently of the classical steroid hormone ligands (reviewed in (105); **Figure 1**). Obesity and T2D may promote features of aggressiveness in ER+ breast cancer, such as elevated proliferation, E2F activation, and growth factor signaling (106); pathways associated with a lack of response to endocrine therapy. In luminal breast tumors and cell lines, evidence shows that activated growth factor receptors can phosphorylate ER and its co-regulatory proteins via hormone-independent mechanisms (107). This ligand-independent activation of ER is often mediated through MAPK and PI3K/AKT signaling pathways that phosphorylate ER on several sites; the most commonly evaluated being S118, a target of MAPK, and S167, a target of AKT (**Figure 1**). Phosphorylation of ER by any pathway can contribute to the transcription of estrogen-responsive genes even in the presence of anti-estrogen therapies (108–110). CAF-derived FGF7 was shown to promote ER phosphorylation and breast cancer cell growth through FGFR2, which ultimately reduced the efficacy of endocrine therapies (111). FGF10/FGFR2 signaling can interfere with classical ER activation to facilitate resistance to endocrine therapies (112). FGF10 was also shown to promote EMT, migration, and colony formation in HR+ and TNBC cells (113). In multiple ER+ breast cancer cell lines, FGF1 and FGF2 can induce membrane ruffling, which often accompanies metastatic invasion (114). A different study showed that nuclear FGFR1 can interact with ER to promote cell proliferation and estrogen-independent transcription of ER target genes (115), showing that some of the effects of FGFR do not rely on FGF ligands. In isolated ER+ breast cancer cells, estrogen treatment was shown to maintain cancer stem cell populations through paracrine FGF9/FGFR3 signaling (116). Likewise, FGFR2 activation maintained mammary tumor-initiating populations in MMTV-PyMT mice, an aggressive model of luminal breast cancer (117). FGFR4, which can mediate signaling from FGF1, FGF4, and FGF8 subfamilies, may facilitate acquired and *de novo* resistance to endocrine therapies in breast cancer (118). Recently, FGF1 was shown to associate with greater phosphorylate of ER at S118, particularly in endocrine-resistant cells that grow in obese mice even after estrogen loss. Elevated pS118 ER associated with shorter recurrence free survival in patients with ER+ tumors (119). Altogether, the effects of FGF and other growth factors on breast cancer cells are broad and involve many different pathways. One potential target of FGFR signaling includes ER activation independently of estrogen ligands. Since the first line therapy for many breast cancer patients involves depleting peripheral estrogen production, it may be worth reconsidering how the tumor environment maintains ER activation through non-classical mechanisms, and whether this occurs more frequently in the context of poor metabolic health.

## 4 Metabolic reprogramming in aggressive breast cancer cells

One mechanism by which obesity and T2D support breast cancer growth and progression may be through metabolic reprogramming that involves alterations in glycolytic and mitochondrial metabolism. As an important hallmark of cancer, metabolic reprogramming allows cancer cells to adapt their increasing energy demands to available resources for growth, motility, proliferation, and function. Aerobic glycolysis has been widely and consistently observed in many cancer types. The rapid ability of cells to convert glucose into pyruvate and lactate can lead to substantial ATP production but can also support biomass generation. Mitochondrial metabolism also generates precursors for fatty acids, nucleotides, and amino acids (120). Several studies have demonstrated that metabolic reprogramming is a prominent feature in therapy resistance and aggressiveness in breast cancer cells. The metabolic milieu that characterizes T2D and often obesity provides a favorable microenvironment to support breast cancer growth, including sustained hyperglycemia and dyslipidemia (121, 122).

Elevated glycolytic metabolism is a feature of aggressive breast cancer, including TNBC and endocrine-resistant ER-positive cancer cells. High levels of tumor glucose uptake (FDG-PET) predicted a shorter progression-free survival interval in patients on endocrine therapy (123). Tamoxifen-resistant human breast cancer cells (LCC2 and LCC9) have greater glycolytic activity than the MCF7 cells from which they were derived and are susceptible to glycolytic inhibition (124). Multiple studies on specific glycolytic enzymes and transporters show the importance of this metabolic pathway in aggressive breast cancer cell behavior. For example, overexpression of the glucose transporter GLUT1 in TNBC cells supports the invasion, migration, and metastatic potential of these cells (125, 126). HK2 expression is associated with tamoxifen resistance in ER+ breast cancer cells (127), and with proliferation of TNBC cells (128). In a recent study, Zhu et al. demonstrated that the transcription factor ETV4 regulates breast cancer stemness and glycolic metabolism by modulating HK activity in both TNBC and ER+ breast cancer cells (129). ENO1, which catalyzes the reversible conversion of 2-phosphoglycerate and phosphoenolpyruvate, has been implicated in tamoxifen resistance by activation of ER and inhibition of apoptosis (130, 131). Lactate production by lactate dehydrogenase (LDH) enzymes is a feature of enhanced glycolytic activity in cancer cells. The LDH enzymes are significantly elevated in TNBC tumors and associate with shorter overall and disease-free survival (132) and with brain metastasis (133). LDH activity is also elevated in cancer-associated adipocytes, an important component of the tumor microenvironment, suggesting a crosstalk that can be promoted by obesity (134). Overexpression of MCT1 and MCT4, monocarboxylate transporters that shuttle lactate across the plasma membrane, strongly associates with worse survival in TNBC and endocrine-resistant breast cancer cells (133, 135). Treatment of tamoxifen-resistant MCF7 cells with FGF1 led to elevated expression of ETV4 and glycolytic genes, including ENO1, LDH, and SLC2A1. Functional evaluation of metabolism after FGF1 treatment demonstrated enhanced glycolysis in endocrine-resistant cells but not oxygen consumption, consistent with metabolic reprogramming and aggressive phenotype (119). Acidification of the tumor microenvironment is suggested to provide multiple advantages including increased angiogenesis, genomic instability, and potentially selecting for apoptosis-resistant tumor clones (136). Overall, altered cancer cell metabolism, particularly a shift to rapid glycolysis and lactate production, is a feature of aggressive breast tumors, whether TNBC or endocrine therapy resistant ER+. Tumors that benefit from or rely on elevated glucose metabolism may thrive in an environment of poor metabolic health in which glucose, as well as other nutrients are readily available, and that also produces growth-promoting signals from multiple cell types in the breast.

### 4.1 EMT and metabolic distinctions of aggressive breast cancer cells

Many potential mechanisms of obesity- or diabetes-associated signaling pathways could stimulate or maintain aggressive cancer cell behavior. One such mechanism may be epithelial to mesenchymal transition (EMT); a feature of aggressive cancer cells that undergo invasion and metastasis. EMT has been linked to altered cancer cell metabolism and to poor metabolic health. The epithelial phenotype is often associated with luminal breast cancers, regardless of ER expression (137). Cells of the basal subtype or TNBC tumors have greater expression of mesenchymal genes, such as SNAI1 (Snail), SNAI2 (Slug), VIM (vimentin), and various MMPs. Multiple studies have suggested that metabolic reprogramming both supports and is caused by the EMT process and related genes (136, 138), and cancer metastasis has been hypothesized to be under metabolic control (139). Several glycolytic enzymes can induce an EMT phenotype, including aldolase A, LDHA, and pyruvate dehydrogenase kinase-1 (136). On the other hand, EMT related proteins may influence metabolic activity in cancer cells (136). One example is a study showing that the Snail transcription factor can inhibit mitochondrial cytochrome C oxidase activity, and may facilitate the loss of fructose-1,6-bisphosphatase 1 in basal cancer cells, which enhances glucose uptake (140). A comparison of metabolic activity between breast cancer cells representing the luminal subtype (MCF7, T47D) and the basal/mesenchymal subtype (MDA-231, MDA-435) revealed distinct metabolic signatures that were influenced by EMT proteins (141). Compared to luminal cells, the basal cells had impaired, but not completely defective mitochondrial function, were less dependent on mitochondrial ATP production, and displayed elevated glycolysis and lactate production. Knockdown of E-cadherin or β-catenin in luminal cells phenocopied the basal cell metabolic phenotype, decreasing mitochondrial respiration and increasing lactate production. Low expression of mitochondrial oxidative genes is associated with metastatic potential and poor clinical outcomes across multiple cancer types (142). In cervical cancer cells, Porporato et al. showed that mitochondria must be present, but defective, in cells undergoing metastasis, based on increased cell migration that accompanied partial electron transport chain inhibition (139). The investigators suggested that metastatic progenitor cells are characterized by mitochondrial superoxide production, both from overloaded TCA cycling and from defective electron transport chain activity (139). Recently, the concept of mitochondrial overload resurfaced with the suggestion that glycolytic production of lactate occurs when cancer cell mitochondria cannot oxidize NADH as fast as glycolysis can produce it (120). It is currently not well understood if aggressive or metastatic breast cancer cells prefer glycolysis because of specific defects in mitochondrial electron transport subunits. A thorough investigation of how mitochondrial function may be altered during breast cancer progression, or in specific populations such as cancer stem cells, may help better target therapies to patients.

### 4.2 The influence of poor metabolic health on EMT and cancer metabolism

Many of the genes encoding glycolytic enzymes are regulated by the HIF1α transcription factor that is upregulated or stabilized in hypoxic environments (143). The consequences to cancer cells include EMT, invasion, metastasis, therapy resistance, and metabolic reprogramming (143). The obese or diabetic breast tissue is often characterized by reduced vascularization, which leads to hypoxia and inflammation. This, coupled with the excess available nutrients is the prime environment to support tumor progression. Sustained hyperinsulinemia in a mouse model of T2D promoted ER+ breast cancer cell growth associated with stabilization of HIF1α (144). A recent study reported that obesity associated with greater DNA damage in the breast epithelium of women with BRCA mutations (76). In several models, DNA damage was shown to be enhanced by estrogen and insulin signaling; two hormones that likely mediate many adverse effects of obesity on breast cancer. The top pathway enriched in isolated breast epithelial organoids from women with obesity and BRCA mutations was HIF1α signaling (76). These two reports clearly link a poor metabolic environment to changes that could facilitate glycolytic metabolism in tumors. Future studies are needed to define the metabolic changes in cancer cells in these contexts, and whether they can be targeted to prevent disease progression.

Within the breast, CAFs and cancer cells have intricate interactions where each can reprogram its metabolism to benefit the other (145). A recent study using murine models of breast cancer found that cancer-associated adipocytes, which are present at the invasive front of tumors, underwent dedifferentiation to a myofibroblast-like cell type that expressed markers of immune cells and ECM remodeling (146). These de-differentiated adipocytes had enhanced glycolytic metabolism and oxygen consumption compared to CAFs that did not arise from adipocytes (146). Preventing adipocyte dedifferentiation reduced tumor growth under chow and high-fat diet conditions (146), indicating the tumor promotional role of lipids when they are present in excess. Another recent study evaluated breast adipose tissue fibroblasts isolated from tissue immediately adjacent to tumors (CAFs) and distant from tumors in the same individuals, classified as either lean (BMI<25kg/m^2^) or obese (BMI>35kg/m^2^) (147). The CAFs from women with obesity expressed higher levels of myoepithelial markers such as CD29, alpha-smooth muscle actin (ACTA2), and connective tissue growth factor (CTGF). In contrast, the CAFs from lean individuals expressed markers associated with inflammation such as IL1β and CXCL10. Notably, FGF2 and to a lesser extent, FGF1, were each more highly expressed in CAFs compared to distant fibroblasts, but the distant fibroblasts from women with obesity had greater FGF2 expression than the lean group. This could indicate a more widespread impact of the tumor on the breast in obesity, or it could reflect a tumor-promotional milieu present throughout the obese breast tissue. In several different assays, the most prominent effect of fibroblast conditioned media on cancer cells, including proliferation and motility, appeared to be from the site of origin, with CAFs imparting a more aggressive cancer cell phenotype than distant fibroblasts. However, compared to all other groups (lean CAFs, lean or obese distant fibroblasts), obese CAFs promoted greater growth of luminal breast cancer spheroids in direct co-culture assays. Interestingly, the pathways altered in ER+/HER2+ (BT474) cancer cells cultured with lean CAFs included cell migration and EMT, while those altered by obese CAFs included cell cycle progression and cellular metabolism. In TNBC (MDA-231), the obese CAFs induced a cancer stem cell-like phenotype, while in ER+/HER2+ cells the impact was greater for EMT (147). Reprogramming of CAF metabolism towards glycolysis and lactate production can promote the growth of TNBC *in vivo* (148). A different study found that extracellular vesicles (EVs) from adipocytes induced genes associated with EMT and cancer stem cells in ER+ and TNBC cell lines (149). Conditioned media from breast adipocytes collected from women with obesity stimulated migration and invasion of breast cancer cells, but this phenotype was attenuated when EVs were depleted (149). TNBC cells grafted in a murine model of streptozotocin-induced diabetes showed greater tumor growth and had elevated expression of EMT markers and glycolytic enzymes compared to cells grown in non-diabetic mice (150). Diet-induced obesity accelerated growth and elevated cancer stem cell and EMT markers in MMTV-Wnt1 mice, a model of TNBC (151). The effects of obesity in this study were mediated by circulating leptin, produced by adipose tissue.

FGFR signaling has been linked to EMT in various cancer types, such as lung, pancreas, and prostate; an effect that may be FGFR isoform-specific (152). In endometrial cancer, FGFR2 mutations were associated with features of EMT, such as high vimentin staining, low E-cadherin, and tumor budding (153). FGFR1 was identified as a potential mediator of EMT-induced drug resistance in EGFR mutant non-small cell lung cancer (154). In HER2-transformed breast cancer cells, FGF2/FGFR1 were identified to be highly expressed in Lapatinib-resistant populations (155). FGF signaling, through the MAPK pathway, stabilized the Twist transcription factor to help maintain the mesenchymal, drug-resistant cell population (155). A separate study showed that, in chemotherapy-resistant MCF7 and MDA-231 cells, glycolytic genes were elevated compared to parental lines, and FGFR4 inhibition reduced glycolytic flux (156). Overexpression of N-cadherin in MMTV-Neu mice, a model of HER2+ breast cancer, did not alter primary tumor latency or growth, but significantly augmented lung metastasis (157). In cell lines derived from these primary tumors, N-cadherin overexpression was associated with greater levels and phosphorylation of FGFR1 and with greater relative levels of the mesenchymal IIIc isoform of FGFR2 compared to the epithelial IIIb isoform. The expression of EMT markers Snail and Slug was elevated in N-cadherin overexpressing cells and was dependent upon FGFR.

Metabolism of other nutrients besides glucose, such as amino acids and lipids, is often altered in aggressive breast tumors. Breast cancer cells are often dependent on glutamine for the production of carbon and nitrogen needed for proliferation, invasion, and metastasis (158–160). Serum glutamine levels are elevated in individuals with obesity and T2D, as well as in breast cancer patients. The amino acid transporters SLC6A14 and SLC1A5 are upregulated in breast cancer cells and implicated in therapy resistance (161). TNBC displays increased glutamine uptake and glutamine-related enzymes (160) and reduction of glutamine transporters reduces the proliferation and migration of TNBC (159). Circulating free fatty acids were correlated with increased proliferation and aggressiveness of ER+ breast cancer via activation of ER and mTOR pathways and reprogrammed cancer cell metabolism (162). In a preclinical model, the induction of hypercholesterolemia in mice resulted in greater breast cancer growth (163). Tamoxifen resistance in ER+ breast cancer cells was found to be linked to deregulation of cholesterol pathways and altered lysosomal integrity (164). ER-dependent breast cancer cells develop resistance to aromatase inhibition through epigenetic and transcriptomic activation of cholesterol biosynthesis that contributes to aggressive breast phenotypes (165). Key enzymes and mediators of fatty acid metabolic pathways have been shown to drive metabolic reprogramming of endocrine-resistant invasive lobular carcinoma cells (166). As more and more investigators incorporate aspects of obesity and metabolic disease into preclinical models and in the analysis of clinical specimens, our understanding of how these environments associate with and influence aggressive tumor cell metabolism will greatly improve.

## 5 Discussion

**Figure 2:**
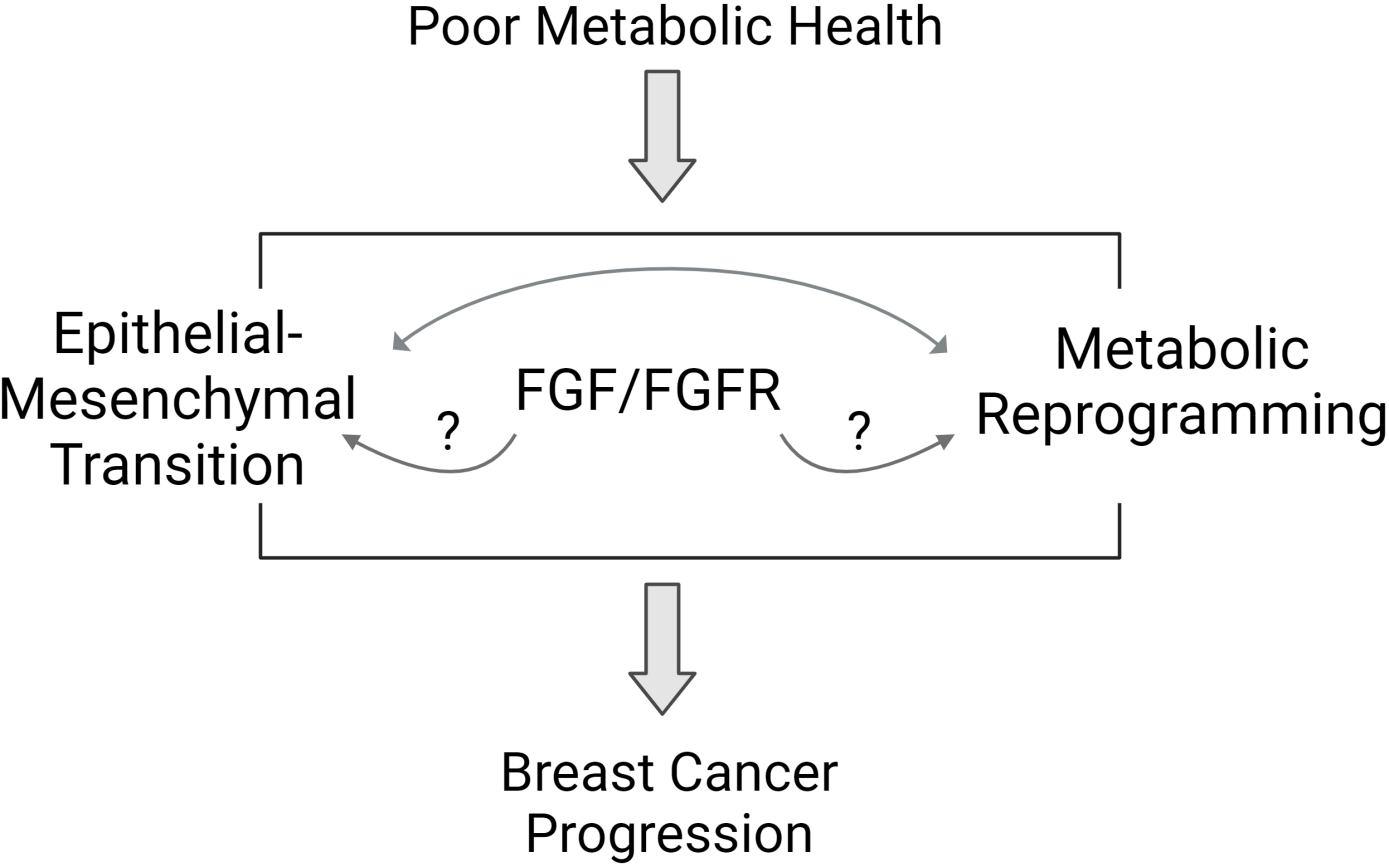
Proposed pathways mediating the link between poor metabolic health and breast cancer biology. Poor metabolic health influences breast cancer progression through a variety of mechanisms. Studies suggest that the FGFR signaling pathway may mediate some of the effects of whole-body metabolism on breast tumor biology. Features of aggressive breast cancer include epithelial to mesenchymal transition (EMT) and metabolic reprogramming. FGFR signaling may influence each of these areas to promote aggressive breast cancer behavior.

Obesity and T2D are closely linked chronic diseases that impact millions of adults worldwide. The proportion of children with obesity has increased in recent decades, foretelling a future where most adults may be impacted by poor metabolic health. The relationship surrounding obesity, T2D, and breast cancer is incredibly complex, but scientists have uncovered many interesting mechanisms. Growth factor signaling from the tumor environment, specifically FGF/FGFR pathways, may be one important link between poor metabolic health and aggressive breast cancer. Cancer cells themselves may benefit from a complex relationship between EMT and metabolic reprogramming that is influenced by host metabolic health through growth factor production and excess nutrient availability (**Figure 2**). People who are metabolically healthy and who do not have obesity do develop and die from breast cancer, but statistically, it is less likely compared to those with poor metabolic health. It is not known if aggressive breast cancers are unique between these two groups of individuals, and currently, BMI and metabolic health is not factored into treatment decisions for breast cancer. Based on the studies reviewed here, some specific knowledge gaps remain. For example, several preclinical studies show a link between FGFR and ER activation. The loss of classical ER target gene expression signature is associated with endocrine therapy resistance, but it isn’t clear if growth factor receptor activation alters the ER-dependent transcriptome resulting in a distinct estrogen-independent ER transcriptome. Is there a gene expression signature that could indicate what is activating ER in endocrine-resistant breast cancers? In some people with obesity or T2D, a strategy to directly target ER may be more effective than targeting estrogen synthesis. Another area for investigation involves the similarities and differences between TNBC and endocrine-resistant ER+ breast cancers. In addition to estrogen-independent ER+ tumor growth, FGFs, and FGFR have been linked to malignant transformation of ER-negative breast cells, potentially accelerating TNBC formation. Are there common driving mechanisms that can be targeted in these tumors specifically? Further, what are the unique molecular responses of endocrine-sensitive and endocrine-resistant breast cancer cells to FGF treatment? From the host perspective, FGF signaling is a normal, necessary growth-promoting pathway, so is it feasible to target these factors directly for prevention or treatment of breast cancer. In summary, the prevalent incidence of obesity, T2D, and poor metabolic health, which may remain undefined, illustrates the importance of paying attention to BMI, adiposity, and individual metabolic state when evaluating breast cancer risk or prognosis. Future research will help illuminate the role of whole-body metabolism in breast cancer incidence and progression.

## Author contributions

All authors listed have made a substantial, direct, and intellectual contribution to the work and approved it for publication.
